# Effect of Forced-Air Solar Drying and Biogas-Assisted Hybrid Solar Drying on the Overall Quality of Dehydrated Andean Berries

**DOI:** 10.3390/foods15203735

**Published:** 2026-10-20

**Authors:** Merbelita Yalta Chappa, Homar Santillan Gomez, Sandra Mori-Vigo, Fernando Isaac Espinoza Canaza, Wildor Gosgot Angeles, Diana Carina Mori Servan, Manuel Oliva-Cruz, Amancio Human Lopez, Carla Ordinola Ramírez, Miguel Ángel Barrena Gurbillón

**Affiliations:** Instituto de Investigación para el Desarrollo Sustentable de Ceja de Selva (INDES-CES), Universidad Nacional Toribio Rodríguez de Mendoza de Amazonas (UNTRM), Chachapoyas 01001, Peru; homar.santillan@untrm.edu.pe (H.S.G.); sandra.mori.epg@untrm.edu.pe (S.M.-V.); fernando.espinoza@untrm.edu.pe (F.I.E.C.); wildor.gosgot@untrm.edu.pe (W.G.A.); diana.mori@untrm.edu.pe (D.C.M.S.); manuel.oliva@untrm.edu.pe (M.O.-C.); humanlopezamancio923@gmail.com (A.H.L.); carla.ordinola@untrm.edu.pe (C.O.R.)

**Keywords:** solar drying, hybrid drying, biogas-assisted drying, Andean berries, physicochemical quality, bioactive compounds

## Abstract

This study evaluated the effect of forced-air solar drying and biogas-assisted hybrid solar drying on the overall quality of dehydrated Andean berries. Blueberry (*Vaccinium corymbosum*) and goldenberry (*Physalis peruviana*) were analyzed before and after dehydration using two systems: forced-air solar drying (DCFA) and biogas-assisted hybrid solar drying (DCFA-BG). Physicochemical properties, instrumental color, texture, rehydration index, total phenolic content, total flavonoid content, and antioxidant capacity were determined. Drying markedly reduced moisture content and water activity in both fruits. In blueberry, DCFA-BG achieved the lowest moisture content (22.00%) and water activity (0.65), whereas goldenberry showed similar final moisture contents under both systems, with lower water activity under DCFA-BG (0.64). Total soluble solids and titratable acidity increased after drying, indicating solute concentration. Color changes were more pronounced in goldenberry, while blueberry showed moderate chromatic variation. Blueberry firmness increased after drying, whereas goldenberry exhibited treatment-dependent textural responses. Blueberry showed greater retention of bioactive properties, with increased antioxidant capacity after drying, whereas goldenberry exhibited substantial reductions in phenolic content, flavonoid content, and antioxidant capacity. Overall, biogas-assisted hybrid solar drying effectively reduced moisture content and water activity, although its effects on product quality depended on the fruit matrix. These findings support its potential application as a sustainable alternative for processing perishable fruits in high-Andean rural agroindustrial contexts.

## 1. Introduction

Berries and berry-like fruits are highly valued food matrices because of their nutritional and functional properties, mainly associated with their content of bioactive compounds such as phenolics, flavonoids, anthocyanins, vitamins, and minerals. These compounds contribute to antioxidant capacity, characteristic pigmentation, and potential health-promoting effects [1,2]. Among these fruits, blueberry (*Vaccinium corymbosum*) is particularly recognized for its high anthocyanin and phenolic content, which has been associated with antioxidant activity and potential health benefits [3,4]. Similarly, goldenberry (*Physalis peruviana*) is valued for its content of vitamin C, carotenoids, flavonoids, and other antioxidant compounds of agrofood interest [5].

However, these fruits are highly perishable due to their high moisture content and water activity. In blueberries, the water content, which is close to 86%, limits shelf life and promotes postharvest losses during storage and commercialization [6,7]. A similar limitation occurs in goldenberry, whose postharvest stability may be affected by changes in texture, color, acidity, and bioactive quality [8,9]. Dehydration represents an effective technological alternative to extend the shelf life of highly perishable fruits. This process reduces water content, limits microbial growth, and decreases physical, chemical, and biochemical reactions associated with product deterioration [10,11]. In addition to improving food stability, dehydration may promote the relative concentration of sugars, organic acids, minerals, and bioactive compounds. Nevertheless, the final quality of dehydrated products depends on the drying method, temperature, airflow, exposure time, and thermal sensitivity of the compounds present in the food matrix [12,13].

In berries, drying can induce relevant changes in physicochemical, bioactive, and textural properties. Although drying may concentrate soluble compounds, it can also promote anthocyanin degradation, loss of lightness, browning, tissue hardening, and reduced rehydration capacity [3]. Solar drying is a sustainable alternative for food preservation, particularly in rural or agro-industrial areas with high solar radiation availability. Compared with open sun drying, solar dryers provide better product protection, improved thermal utilization, and reduced risk of external contamination [14]. Within these systems, forced airflow improves heat and mass transfer during dehydration; continuous air circulation facilitates moisture removal from the product surface and can reduce drying time, thereby increasing process efficiency [15,16]. Despite these advantages, solar drying is limited by variations in solar radiation, ambient temperature, relative humidity, and cloudiness. These conditions can cause thermal fluctuations inside the drying chamber and affect process uniformity, particularly when heat-sensitive products require prolonged drying periods [17,18].

Biogas-assisted hybrid solar drying systems have emerged as an alternative to complement solar energy input. The use of biogas as an auxiliary heat source can help maintain more stable thermal conditions during drying, especially during periods of low solar radiation, while also integrating the energetic use of organic residues into agro-industrial processes. However, the incorporation of an auxiliary heat source should not be evaluated only from an energetic perspective. It is also necessary to determine its effect on the overall quality of the final product, since higher temperatures or longer exposure times may modify color, texture, acidity, and antioxidant activity of dehydrated fruits [19,20].

Although several studies have evaluated fruit and berry dehydration using different drying methods, limited information is available on the comparison between forced-air solar drying and biogas-assisted hybrid solar drying in Andean berries. This knowledge gap is relevant because the selection of a drying technology should consider not only moisture reduction, but also the preservation of physicochemical, bioactive, and textural properties. Therefore, this study aimed to evaluate the effect of forced-air solar drying and biogas-assisted hybrid solar drying on the overall quality of dehydrated Andean berries, using *Vaccinium corymbosum* and *Physalis peruviana* as food matrices.

## 2. Materials and Methods

### 2.1. Study Area

The study was conducted on the campus of the Universidad Nacional Toribio Rodríguez de Mendoza de Amazonas (UNTRM), located in the city of Chachapoyas, province of Chachapoyas, Amazonas region, Peru. The solar drying systems were installed in the experimental area of the Research Center for Climatology and Alternative Energies Project (PROCICEA).

During the dehydration trials, external environmental conditions, including global solar radiation and ambient temperature, were recorded using a DAVIS Vantage Connect weather station belonging to the Research Institute for the Sustainable Development of Ceja de Selva (INDES-CES) of UNTRM. These records were used to characterize the climatic conditions under which the solar drying systems operated.

**Figure 1:**
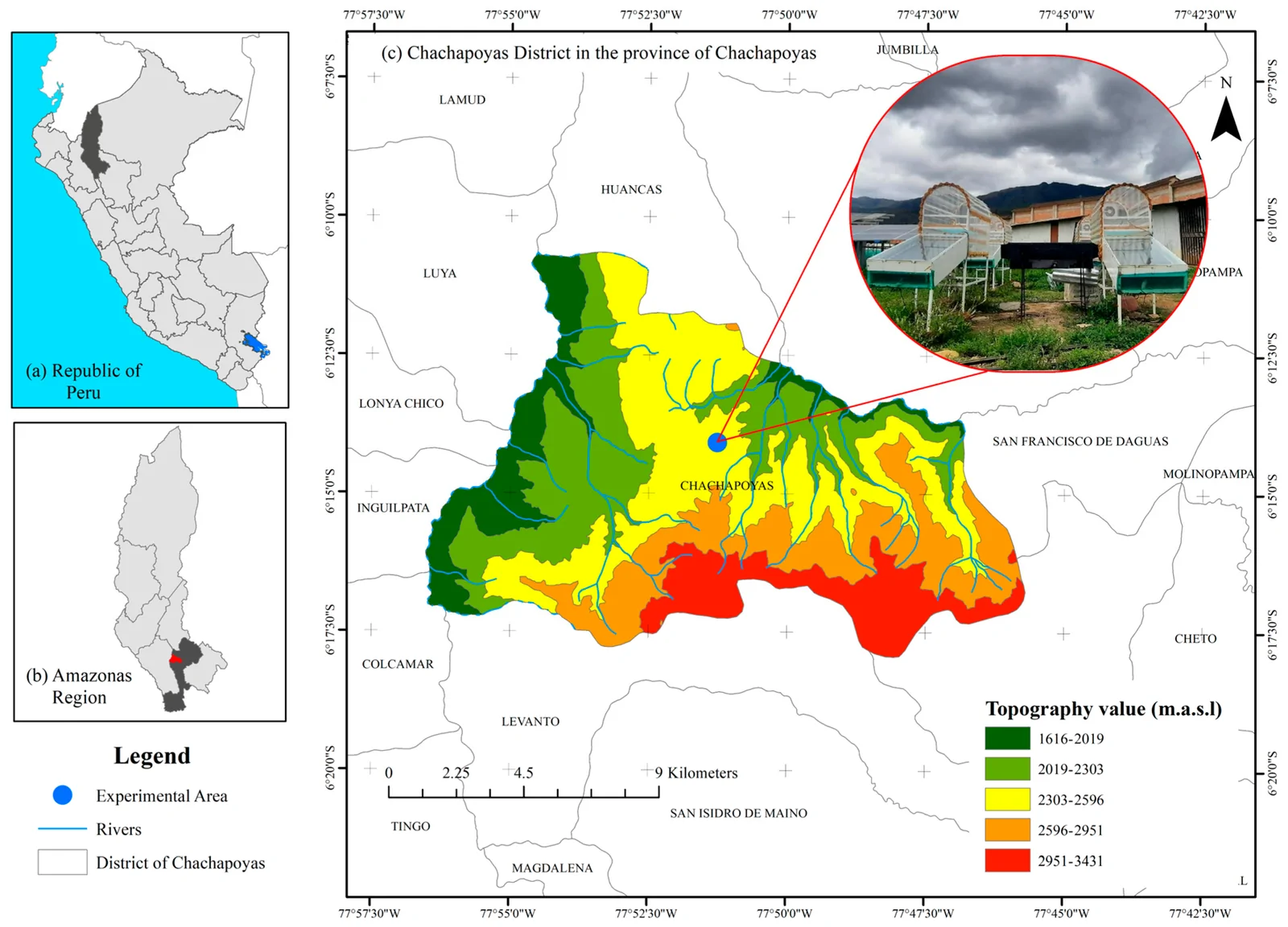
Geographic location of the solar dryers equipped with a solar collector.

The exact location of the systems corresponded to the coordinates 6°13′54.1″ S and 77°52′08.5″ W, equivalent to −6.2317° and −77.8690° in decimal coordinates (Figure 1). Chachapoyas is characterized by a marked climatic seasonality determined by the rainfall regime, with a rainy season from October to March and a dry season from April to September [21,22].

### 2.2. Experimental Design

The study was conducted under a completely randomized design with a 2 × 3 factorial arrangement (Table 1). The evaluated factors were fruit species and treatment condition. The first factor included two species: blueberry (*Vaccinium corymbosum*) and goldenberry (*Physalis peruviana*). The second factor included three treatment conditions: fresh non-dehydrated fruit (FSD), forced-air solar drying (DCFA), and biogas-assisted hybrid solar drying (DCFA-BG).

Fresh non-dehydrated fruit was used as the control to establish the initial quality characteristics of each species. The DCFA and DCFA-BG treatments were used to evaluate the effects of the drying systems on the physicochemical, bioactive, textural, and rehydration properties of the dehydrated fruits.

Physicochemical variables included pH, titratable acidity, total soluble solids, moisture content, water activity, and instrumental color. Bioactive variables included total phenolic content, total flavonoid content, and antioxidant capacity. In addition, firmness and rehydration index were evaluated.

Physicochemical, bioactive, textural, and rehydration analyses were performed in triplicate for each treatment. The rehydration index was determined only in dehydrated samples. External environmental conditions, such as global solar radiation and ambient temperature, were recorded to characterize the operational context of the drying systems, but they were not considered controlled experimental factors.

### 2.3. Solar Dehydration Systems

Two solar dehydration systems with similar structural configurations were used: a forced-air solar dryer (DCFA) and a biogas-assisted forced-air hybrid solar dryer (DCFA-BG). Both systems consisted of a flat-plate solar collector, a dehydration chamber, and internal trays for sample distribution.

The solar collector was composed of a steel absorber plate measuring 1050 × 600 × 2 mm, painted black to improve solar energy absorption. The plate was thermally insulated with 20 mm thick expanded polystyrene and covered with aluminum foil to reduce heat losses. The collector glazing consisted of 5 mm thick single glass, with an approximate distance of 150 mm between the glazing and the absorber plate.

The dehydration chamber measured 1200 × 600 × 900 mm and was covered with 2 mm thick translucent corrugated polycarbonate. Inside the chamber, trays made of nylon mesh with openings smaller than 5 mm were used to ensure uniform fruit distribution and to allow the passage of hot air during the drying process.

The DCFA system operated through forced air circulation generated by a 220–240 V axial extractor powered by photovoltaic energy. During extractor operation, the average air mass flow rate was approximately 0.038 kg·s^−1^. The DCFA-BG system maintained the same structural configuration as the DCFA system but incorporated an auxiliary heat source through biogas combustion. This thermal assistance complemented the solar energy input during dehydration; therefore, the system was considered a hybrid solar dryer. The technical characteristics of both solar dehydration systems are summarized in Table 2.

**Figure 2:**
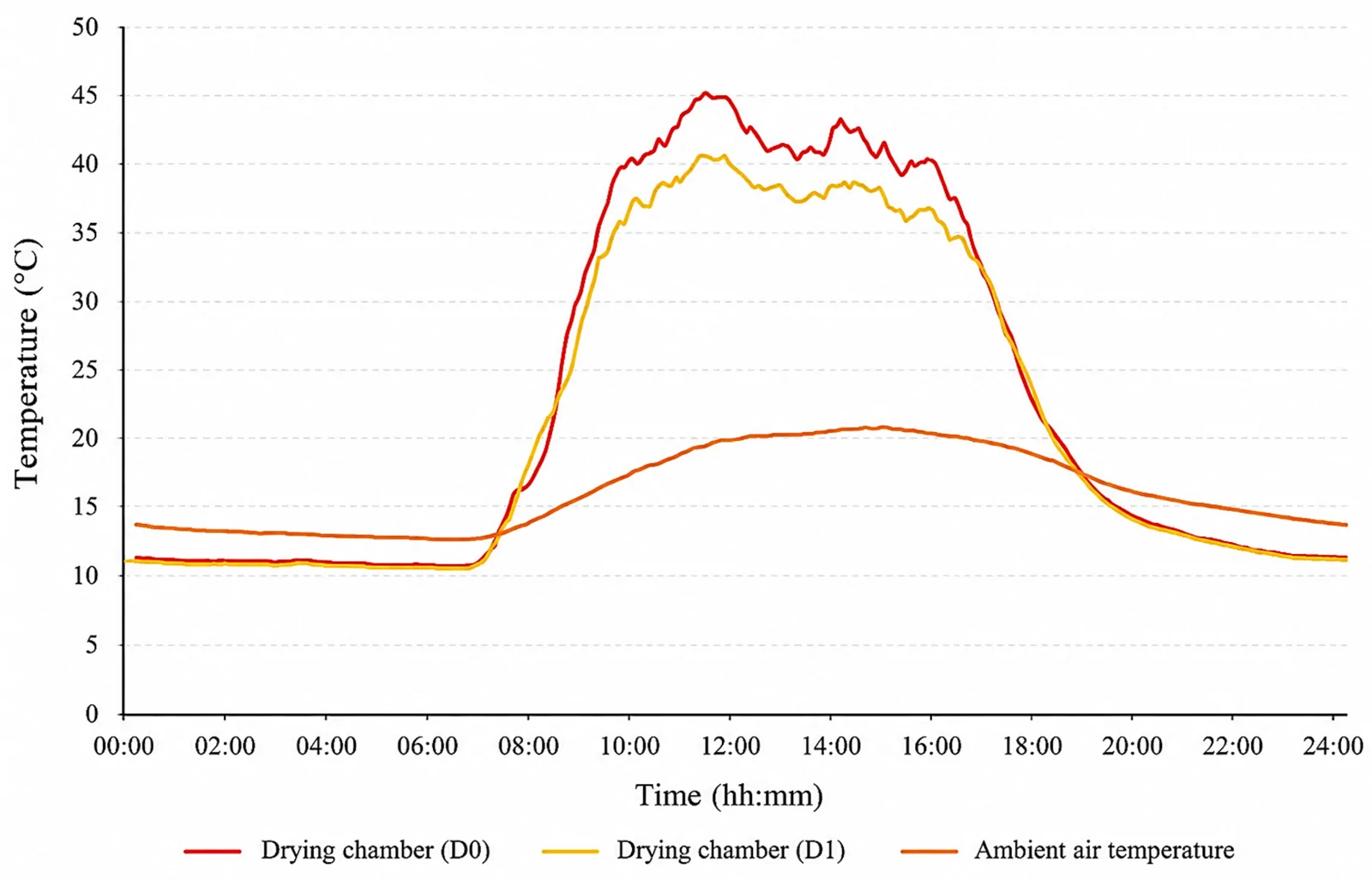
Representative temperature profile of the forced-air solar drying system (DCFA) during the drying process. Temperatures were monitored inside the drying chamber and compared with ambient air temperature.

**Figure 3:**
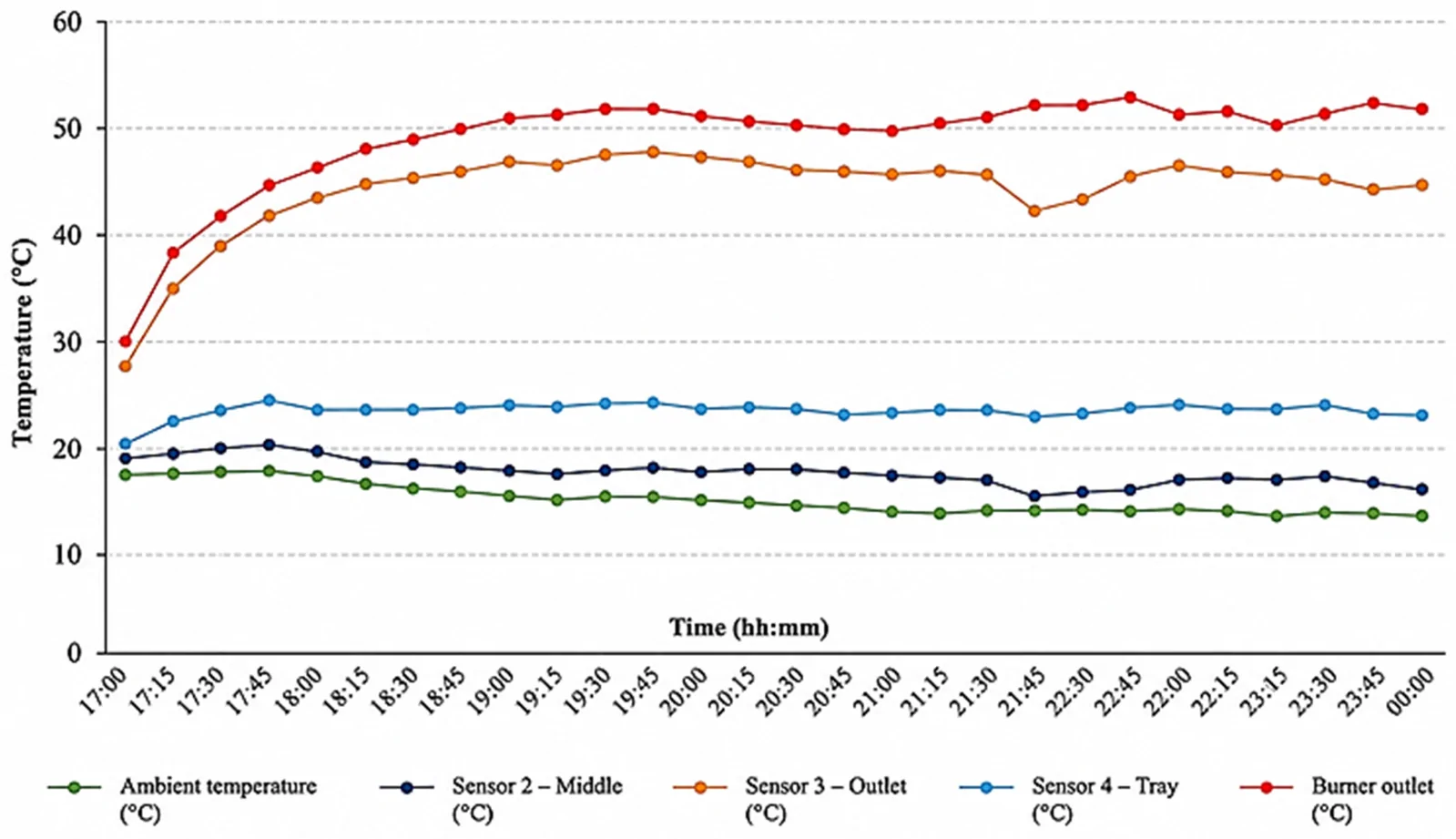
Representative temperature profiles recorded at different locations of the DCFA-BG hybrid solar drying system during nocturnal operation. Air temperature was monitored at the burner outlet, drying tray, collector outlet, collector middle section, and ambient environment.

System temperature was monitored using DS18B20 sensors connected to an Arduino MEGA 2560 module. The sensors were installed at representative locations throughout the system, including the collector inlet, middle section of the collector, transition zone between the collector and drying chamber, and inside the drying chamber. Temperature data were recorded at 5-min intervals throughout the drying process. Representative temperature profiles of the DCFA and DCFA-BG systems are presented in Figure 2 and Figure 3, respectively, to characterize the thermal conditions during dehydration. These temperature profiles were used to support the interpretation of the physicochemical, bioactive, textural, and rehydration responses of the dehydrated fruits.

### 2.4. Raw Material, Selection, and Sample Preparation

Fresh blueberry (*Vaccinium corymbosum*) and goldenberry (*Physalis peruviana*) fruits were used as food matrices to evaluate the solar dehydration systems. The fruits were purchased from the Modelo Market in the city of Chachapoyas, Amazonas, Peru, from local commercial vendors, and immediately transported to the Cocoa Quality Control Laboratory of the Research Institute for the Sustainable Development of Ceja de Selva, Universidad Nacional Toribio Rodríguez de Mendoza de Amazonas. No formal taxonomic identification was performed, and herbarium voucher specimens were not prepared, as the study was conducted using commercially available fruits. The fruits were processed on the same day of purchase to minimize quality changes prior to the dehydration experiments.

The fruits were manually selected according to commercial maturity and visual quality criteria, considering uniform color, homogeneous size, characteristic firmness, and absence of mechanical damage, dehydration symptoms, rot, or visible microbial deterioration. The average initial fruit weights were 2.3 g for *V. corymbosum* and 10.6 g for *P. peruviana*.

Before dehydration, the fruits were washed with potable water and disinfected by immersion in a 0.5% sodium hypochlorite solution for 5 min. The samples were then drained at room temperature to remove excess surface solution.

After conditioning, the fruits were randomly assigned to the corresponding experimental treatment: fresh non-dehydrated fruit, forced-air solar drying, or biogas-assisted hybrid solar drying. Fresh samples were analyzed as the initial control, whereas samples assigned to the DCFA and DCFA-BG treatments were uniformly distributed on the trays of the solar dehydration systems.

### 2.5. Dehydration Process of Andean Berries

The conditioned samples of *V. corymbosum* and *P. peruviana* were uniformly distributed on the mesh-bottom trays of the solar dehydration systems. The fruits were arranged without overlapping to ensure homogeneous exposure to the hot airflow and to improve heat and mass transfer during the drying process.

Dehydration was carried out during the daytime period, from 08:00 to 17:00 h, under real environmental operating conditions. Since both species required more than one day to reach mass stability, the samples remained inside the dehydration chamber throughout the experimental period, maintaining continuous exposure to the microclimate generated by each drying system.

Fruit mass loss was monitored during the process using a digital balance with a precision of ±0.01 g. Weight measurements were recorded every 10 min to evaluate the progressive reduction in moisture during dehydration. The samples were removed from the drying systems when the variation in weight between consecutive measurements was minimal, indicating a tendency toward process equilibrium.

At the end of solar dehydration, the samples were placed in an oven at 90 °C until constant weight was reached. This value was considered the dry solid mass and was subsequently used for moisture content calculations and characterization of the dehydrated product.

The same operational procedure was applied to both drying systems, DCFA and DCFA-BG, allowing comparison of the effect of biogas thermal assistance on the final quality of the dehydrated berries.

### 2.6. Physicochemical Characterization

Physicochemical characterization was performed on fresh and dehydrated fruits of *V. corymbosum* and *P. peruviana*. The analyses were carried out using whole fruits previously washed and disinfected, maintaining the integrity of the food matrix used in the dehydration process. For analyses requiring extraction or dilution, representative portions of the whole fruit were used without separating structural components. All determinations were performed in triplicate for each treatment.

#### 2.6.1. pH Determination

The pH was determined from an aqueous suspension prepared with 3 g of sample and 45 mL of distilled water. The mixture was homogenized for 1 h using an orbital shaker and then filtered through Whatman filter paper. The pH reading was performed using a previously calibrated digital potentiometer (HORIBA Scientific LAQUA PH1300, Kyoto, Japan) with standard buffer solutions, according to the procedure described by [23].

#### 2.6.2. Titratable Acidity

Titratable acidity was determined by acid–base titration. A 10 mL aliquot of the previously prepared aqueous extract was titrated with 0.1 N NaOH using phenolphthalein as an indicator until a persistent pale pink color was observed [24]. The results were expressed as grams of citric acid per 100 g of sample using the following equation:

(1) $$Titratable\;acidity\;\left(\%\right)=\frac{V\times N\times M}{P}\times 100\%$$

Here, the following applies:

V = Volumen of NaOH used (mL)

N = Normality of NaOH (N)

M = milliequivalent factor of citric acid (meq/L)

P = sample weight (g)

#### 2.6.3. Total Soluble Solids

Total soluble solids were determined using a digital refractometer previously calibrated with distilled water. For fresh fruits, the juice extracted from the pulp was used directly, whereas for dehydrated samples, the aqueous extract prepared with 3 g of sample and 45 mL of distilled water was used; the results were expressed as °Brix [25].

#### 2.6.4. Moisture Content

Moisture content was determined using the gravimetric oven-drying method. Approximately 3 g of sample was weighed in previously dried and tared crucibles. The samples were dried at 105 °C until constant weight was reached. Subsequently, the crucibles were cooled in a desiccator and weighed again. Moisture content was calculated using the following equation:

(2) $$Moisture\;content\;\left(\%\right)=\frac{W_{1}-W_{2}}{W_{1}}\times 100\%$$

Here, the following applies:

$W_{1}$ = weight of the fresh sample (g)

$W_{2}$ = weight of the dried sample (g)

#### 2.6.5. Water Activity

Water activity was determined using a portable water activity meter. For each determination, 1 g of sample was placed in the measurement chamber, ensuring uniform distribution to avoid empty spaces that could affect the reading. Measurements were performed in triplicate, and the results were expressed as water activity values $(a_{w}$) [26,27].

#### 2.6.6. Instrumental Color

The color of fresh and dehydrated samples was determined using a colorimeter, and the parameters of the CIELAB color space were recorded: lightness (L*), red–green coordinate (a*), and yellow–blue coordinate (b*). The total color difference between fresh and dehydrated fruits was calculated using the following according to Equation (3), and the Whiteness Index was calculated using Equation (4).

(3) $$∆E=\sqrt{{\left(L^{*}-L_{0}\right)}^{2}+{\left(a^{*}-a_{0}\right)}^{2}+{\left(b^{*}-b_{0}\right)}^{2}}$$

Here, the following applies:

$L^{*},a^{*},b^{*}$ = using the fruit color values as reference

$L_{0}$, $a_{0}$, $b_{0}$ = color values of fresh fruit (reference)

(4) $$WI=100-\sqrt{{\left(100-L^{*}\right)}^{2}+{\left(a^{*}\right)}^{2}+{\left(b^{*}\right)}^{2}\;}$$

### 2.7. Determination of Texture Profile and Rehydration Index

#### 2.7.1. Texture Profile

The texture profile was evaluated in fresh and dehydrated fruits of *V. corymbosum* and *P. peruviana* using a CT3 texture analyzer (Brookfield Engineering Labs, Inc., Middleborough, MA, USA) equipped with a 10 kg load cell and a TA44 probe.

Measurements were performed on whole fruits previously conditioned according to each treatment. A compression test was applied using a pre-test speed of 1 mm·s^−1^, a trigger force of 5 g, 75% compression, a waiting time of 0 s, a test speed of 5 mm·s^−1^, and a post-test speed of 5 mm·s^−1^. Firmness (N), adhesiveness (mJ), and deformation (%) were obtained from the test. Measurements were performed in triplicate for each treatment [28].

#### 2.7.2. Rehydration Index

The rehydration index was determined only in dehydrated samples, as this parameter evaluates the ability of the dried product to absorb water after dehydration. For each analysis, 1 g of dehydrated sample was immersed in distilled water at room temperature (25 °C). The sample was then removed, filtered using Whatman No. 42 filter paper to eliminate excess surface water, and weighed again. The immersion, filtration, and weighing procedure was repeated until constant weight was reached.

The rehydration index was calculated using the following Equation (5):

(5) $$IR=\frac{W_{A}}{W_{B}}$$

Here, the following applies:

$W_{B}$ = peso de la muestra antes de la rehidratación (g)

$W_{A}$ = peso de la muestra después de la rehidratación (g)

### 2.8. Determination of Bioactive Properties

#### 2.8.1. Preparation of the Methanolic Extract

The extraction of bioactive compounds was performed from fresh and dehydrated fruits of *V. corymbosum* and *P. peruviana*. For fresh samples, 1 g of fresh fruit was used, whereas 100 mg of dehydrated fruit was used for dehydrated samples. The different sample weights were selected to account for the higher concentration of dry matter in dehydrated fruits, allowing the preparation of extracts within the optimal analytical range for the spectrophotometric determination of total phenolics, flavonoids, and antioxidant capacity. In both cases, the samples were mixed with 10 mL of 80% methanol [29]. The mixtures were shaken for 1 h and then centrifuged at 3000 rpm for 15 min. The supernatant was filtered through Whatman No. 42 filter paper and transferred to Falcon tubes for subsequent analysis of total phenolic content, total flavonoid content, and antioxidant capacity.

#### 2.8.2. Total Phenolic Content

Total phenolic content was determined using the Folin–Ciocalteu method. Briefly, 20 μL of extract was mixed with 40 μL of 10% Folin–Ciocalteu reagent. After 5 min of reaction, 160 μL of 7.5% sodium carbonate solution was added.

The mixture was incubated in the dark for 60 min at room temperature. Absorbance was then measured at 765 nm using a microplate spectrophotometer. Quantification was performed using a gallic acid calibration curve in the range of 0–140 mg/L, with the regression equation (y = 0.005x − 0.019) and (R^2^ = 0.991) [30].

The results were expressed as milligrams of gallic acid equivalents per gram of sample (mg GAE/g).

#### 2.8.3. Total Flavonoid Content

Total flavonoid content was determined using the aluminum chloride colorimetric method. For the analysis, 20 μL of extract was mixed with 136 μL of 30% ethanol. The mixture was allowed to stand for 5 min, and then 10 μL of 0.5 mol/L NaNO_2_ and 10 μL of 0.3 mol/L AlCl_3_ were added. After a further 5 min incubation, 40 μL of 1 M NaOH was added.

Absorbance was measured at 510 nm using a microplate spectrophotometer. Quantification was performed using catechin as the standard, with a calibration curve ranging from 0 to 105 mg/L, a regression equation of (y = 163.6847x + 0.6420), and (R^2^ = 0.998) [31].

The results were expressed as milligrams of catechin equivalents per gram of sample (mg CAT/g).

#### 2.8.4. Antioxidant Capacity by DPPH Assay (2,2-Difenil-1-picrilhidrazilo)

Antioxidant capacity was determined using the DPPH radical scavenging assay. For this purpose, 20 μL of extract was mixed with 130 μL of 0.1 mM DPPH solution prepared in methanol. The mixtures were incubated in the dark for 30 min at room temperature. Absorbance was then measured at 525 nm using a microplate spectrophotometer.

Antioxidant capacity was quantified using a Trolox calibration curve, and the results were expressed as micromoles of Trolox equivalents per gram of sample (µmol TE/g) [32].

The percentage of DPPH radical inhibition was calculated using the following Equation (6):

(6) $$\%\;de\;inhibición=\frac{{Abs}_{control}-{Abs}_{muestra}}{{Abs}_{control}}\times 100$$

### 2.9. Statistical Analysis

The data obtained were processed using descriptive and inferential statistics. Results were expressed as mean ± standard deviation. Data normality was assessed using the Shapiro–Wilk test. When the data met the assumptions of normality, analysis of variance was applied, followed by Tukey’s multiple comparison test. When the data did not meet parametric assumptions, the Kruskal–Wallis non-parametric test was used. All statistical analyses were performed using IBM SPSS Statistics v.26. Differences were considered statistically significant at *p* < 0.05.

## 3. Results and Discussion

### 3.1. Thermal Conditions During Drying

The representative temperature profiles of the DCFA and DCFA-BG systems (Figure 2 and Figure 3) indicate that both drying systems operated under different thermal conditions throughout the dehydration process. While the DCFA system depended exclusively on solar energy, the DCFA-BG system maintained a more stable thermal regime due to the auxiliary heat supplied by biogas, particularly during periods of reduced solar radiation. Consequently, the differences observed in the physicochemical, bioactive, textural, and rehydration properties of the dehydrated fruits should be interpreted considering the temperature variations, thermal stability, and exposure conditions associated with each drying system. Table 3 presents the pH, titratable acidity, and total soluble solids of *V. corymbosum* and *P. peruviana* in the fresh state and after drying using the DCFA and DCFA-BG systems.

### 3.2. pH, Titratable Acidity, and Total Soluble Solids

Table 3 shows the pH, titratable acidity, and total soluble solids of *V. corymbosum* and *P. peruviana* in the fresh state and after drying using the DCFA and DCFA-BG systems. Overall, both dehydration systems modified the chemical characteristics of the fruits, although the magnitude of these changes depended on the fruit species and the drying system applied.

In *V. corymbosum*, pH decreased from 3.50 in the fresh fruit to 3.00 under DCFA, whereas DCFA-BG resulted in a higher value (3.70). This response suggests that the drying system influenced the active acidity of the product. The lower pH observed after DCFA may be associated with the concentration of organic acids during moisture removal, whereas the slightly higher pH under DCFA-BG could reflect thermal transformations of acidic compounds or changes in their availability within the dehydrated matrix.

In *P. peruviana*, pH remained unchanged after DCFA (3.88) but increased to 4.15 under DCFA-BG. Despite these differences, all treatments remained within the acidic range, which contributes to limiting the growth of many spoilage microorganisms and favors the preservation of dehydrated fruit products.

Titratable acidity increased markedly after dehydration in both species. In *V. corymbosum*, acidity increased from 1.00 to 3.50–3.60 g/100 g, whereas in *P. peruviana* it increased from 1.92 to 4.63 g/100 g under DCFA and reached 8.50 g/100 g under DCFA-BG. These increases are mainly attributable to the concentration of organic acids resulting from water removal, although thermal transformations and redistribution of acids within the fruit matrix may also have contributed.

Total soluble solids increased substantially after drying in both fruits. In *V. corymbosum*, TSS increased from 11.20 to 36.70 and 33.40 °Brix under DCFA and DCFA-BG, respectively, whereas in *P. peruviana*, values increased from 13.90 to 43.92 and 32.94 °Brix. This increase reflects the concentration of sugars, organic acids, and other soluble constituents as moisture was removed during dehydration.

The higher TSS values observed in *P. peruviana* suggest a greater concentration of soluble compounds after drying. The differences between drying systems indicate that drying dynamics and thermal conditions influenced the measured soluble solids content. Overall, the observed changes in pH, titratable acidity, and TSS demonstrate that both drying systems modified the physicochemical properties of the fruits, with the magnitude of these effects depending on the fruit species and drying technology.

### 3.3. Moisture Content and Water Activity

Table 4 presents the moisture content and water activity of *V. corymbosum* and *P. peruviana* in the fresh state and after solar drying treatments. In both species, the DCFA and DCFA-BG systems markedly reduced moisture content and water activity compared with the fresh samples, demonstrating the effectiveness of the applied dehydration processes.

In *V. corymbosum*, moisture content decreased from 84.95% in fresh fruit to 36.74% under DCFA and 22.00% under DCFA-BG, indicating greater water removal by the hybrid drying system. Similarly, water activity decreased from 0.82 to 0.73 and 0.65 under DCFA and DCFA-BG, respectively. These results suggest that the complementary heat supplied by the biogas-assisted hybrid system promoted more efficient dehydration and reduced the amount of free water available for microbial growth and deterioration reactions, thereby improving product stability.

In *P. peruviana*, moisture content decreased from 80.38% in fresh fruit to 24.79% under DCFA and 25.16% under DCFA-BG, indicating that both drying systems achieved comparable dehydration efficiency. Water activity also declined from 0.79 to 0.67 and 0.64, respectively, with DCFA-BG producing slightly lower values. These findings suggest that the drying response in goldenberry depended not only on the drying technology but also on fruit structural characteristics that influence water migration and retention during dehydration.

The substantial reduction in moisture content observed in both species confirms the effectiveness of the evaluated solar drying systems. During dehydration, heated air increases the vapor pressure gradient between the fruit matrix and the surrounding environment, promoting moisture diffusion and evaporation [8]. The superior performance of DCFA-BG in *V. corymbosum* suggests that biogas thermal assistance improved thermal stability within the drying chamber, reducing the effects of fluctuations in solar radiation and enhancing water removal efficiency [15].

The similar final moisture content obtained in *P. peruviana* under both drying systems indicates that water removal was influenced not only by the drying conditions but also by intrinsic fruit characteristics such as skin properties, soluble solids content, and internal water distribution. From a technological perspective, water activity is more relevant than total moisture because it determines the amount of water available for microbial growth and deterioration reactions. Although reducing $a_{w}$ improves product stability, values above 0.60 may still permit the survival of osmophilic microorganisms under favorable storage conditions. Therefore, appropriate packaging and storage conditions remain essential to ensure product stability [33,34].

Overall, both drying systems effectively reduced moisture content and water activity, thereby improving the physicochemical stability of the dehydrated berries. DCFA-BG generally achieved the lowest water activity values, particularly in *V. corymbosum*, suggesting a technological advantage for extending product shelf life. Nevertheless, greater moisture removal should be balanced with the preservation of quality attributes such as color, texture, rehydration capacity, and bioactive compounds. Because the final water activity values remained close to or above 0.64, appropriate packaging, humidity control during storage, and microbiological evaluation are recommended to ensure product stability and safety. Therefore, optimization of drying conditions should be species-specific to achieve an appropriate balance between product stability and quality [3].

### 3.4. Color

Table 5 shows the instrumental color parameters (L*), (a*), (b*), and the total color difference (Δ*E*) of *V. corymbosum* and *P. peruviana* in the fresh state and after drying treatments. Overall, both DCFA and DCFA-BG modified the color of the evaluated fruits, although the magnitude of the change was more pronounced in *P. peruviana*.

In *V. corymbosum*, lightness (L*) showed moderate variation among treatments. The value decreased from 25.67 in fresh fruit to 22.34 under DCFA, indicating slight darkening of the dehydrated fruit. In contrast, theDCFA-BG treatment showed an (L*) value of 26.45, similar to or slightly higher than that of the fresh sample. This behavior suggests that the chromatic response of blueberry depended on the interaction between the drying system and the stability of the pigments present in the fruit matrix.

The (a*) coordinate, associated with reddish tones, increased in *V. corymbosum* after drying, particularly under DCFA. Similarly, the (b*) coordinate changed from negative values in the fresh fruit to positive values in the dehydrated samples, indicating a reduction in the bluish characteristic of fresh blueberry and a shift toward warmer color tones. These changes may be related to partial degradation or transformation of anthocyanins and other heat-sensitive pigments during drying.

In *P. peruviana*, color changes were more evident. Lightness decreased from 63.49 in the fresh fruit to 36.83 under DCFA and 34.75 under DCFA-BG, indicating substantial darkening after dehydration. Likewise, the (b*) coordinate, associated with yellow tones, decreased from 53.56 to 28.54 and 30.80 under DCFA and DCFA-BG, respectively. This reduction reflects a loss of the intense yellow hue characteristic of fresh goldenberry.

The decrease in (L*) and (b*) in *P. peruviana* may be attributed to browning reactions, pigment concentration, carotenoid degradation, and non-enzymatic reactions induced by heat. In fruits rich in sugars and organic acids, drying can promote Maillard reactions and caramelization, especially when exposure time and thermal intensity increase during progressive moisture reduction [35].

The total color difference (Δ*E*) quantified the magnitude of visual change relative to the fresh fruit. In *V. corymbosum*, Δ*E* values were 4.51 under DCFA and 4.90 under DCFA-BG, indicating perceptible color changes after drying. In *P. peruviana*, the values were considerably higher, reaching 27.63 under DCFA and 37.47 under DCFA-BG, confirming that goldenberry was more sensitive to drying-induced chromatic changes.

The total color difference (Δ*E*) provides an instrumental measure of chromatic variation relative to fresh fruit. The values obtained indicate that both drying systems modified the color characteristics of the fruits, with more pronounced changes in *P. peruviana*. These findings highlight the importance of controlling drying conditions to preserve the instrumental color properties of dehydrated products [36,37].

When comparing the drying systems, DCFA-BG produced slightly higher (Δ*E*) values in both species, particularly in *P. peruviana*. This suggests that biogas thermal assistance, while contributing to moisture and water activity reduction, may intensify chromatic changes when the fruit matrix is more heat-sensitive. Therefore, the use of the hybrid system should be optimized to balance drying efficiency, product stability, and visual quality preservation.

### 3.5. Texture Profile and Rehydration Index

Table 6 presents the firmness and rehydration index of *V. corymbosum* and *P. peruviana* in the fresh state and after solar drying treatments. In both species, dehydration modified fruit firmness, indicating structural changes associated with moisture loss and shrinkage of plant tissue.

In *V. corymbosum*, firmness increased from 2.89 N in fresh fruit to 10.14 N under DCFA and 10.89 N under DCFA-BG. This increase indicates that both drying systems promoted greater mechanical resistance in blueberry, probably as a consequence of water loss, soluble solid concentration, and cellular matrix contraction during dehydration.

In *P. peruviana*, firmness also increased after drying, although differences were observed between systems. The DCFA treatment reached 9.02 N, whereas DCFA-BG showed a lower value of 6.89 N. This suggests that forced-air solar drying produced a more rigid structure in goldenberry, while biogas-assisted hybrid drying may have generated a less hardened product, possibly due to differences in heat distribution, moisture migration, and tissue contraction during drying.

The increase in firmness observed in dehydrated fruits is consistent with the physical changes expected during drying. As moisture content decreases, plant tissues lose turgor, shrink, and may undergo partial cellular collapse, increasing resistance to compression. Similar behavior has been reported in dehydrated fruits, where water removal modifies the texture and elasticity of the final product [28,38].

The textural response depended on the fruit species. In *V. corymbosum*, both drying systems produced similar firmness values, indicating that blueberry exhibited a comparable structural response to DCFA and DCFA-BG. In contrast, *P. peruviana* showed higher firmness under DCFA than under DCFA-BG, suggesting that goldenberry tissue was more sensitive to the operating conditions of each drying system.

The rehydration index was used to evaluate the ability of dehydrated samples to absorb water after drying. In *V. corymbosum*, the rehydration index was higher under DCFA, with a value of 2.17, compared with 1.94 under DCFA-BG. This result suggests that forced-air solar drying may have preserved an internal structure slightly more favorable for water uptake during rehydration. In *P. peruviana*, the rehydration index was slightly higher under DCFA-BG, reaching 2.13, compared with 1.91 under DCFA. This indicates that biogas-assisted thermal support did not necessarily reduce the rehydration capacity of goldenberry. Instead, its effect depended on the fruit matrix and on how tissue structure responded to the drying conditions.

Rehydration capacity is closely related to the degree of structural damage produced during drying. A higher rehydration index is usually associated with a less collapsed matrix, greater porosity, or lower irreversible tissue damage. Conversely, lower values may indicate greater shrinkage, hardening, or reduced water absorption capacity after dehydration [19,39]. These findings confirm that the selection of a drying system should consider not only moisture reduction, but also structural preservation and the potential use of the dehydrated product for direct consumption or rehydrated applications.

### 3.6. Bioactive Properties and Antioxidant Capacity

Table 7 presents the total phenolic content, total flavonoid content, and antioxidant capacity of *V. corymbosum* and *P. peruviana* in the fresh state and after solar drying treatments. The results showed species-dependent responses, indicating that the effect of drying on bioactive compounds depends on both the fruit matrix and the dehydration system applied.

In *V. corymbosum*, total phenolic content decreased from 152.00 mg GAE/g in fresh fruit to 142.00 mg GAE/g under DCFA and 128.20 mg GAE/g under DCFA-BG. This reduction was more evident in the biogas-assisted hybrid system, suggesting that greater thermal exposure or more intense drying conditions may have partially affected the stability of phenolic compounds. Phenolics in berries may degrade during drying due to temperature, oxygen exposure, and processing time, although the extent of degradation depends on fruit structure and operating conditions [3].

Total flavonoid content in *V. corymbosum* did not show significant differences among treatments. Values were 107.64 mg CAT/g in fresh fruit, 114.85 mg CAT/g under DCFA, and 105.87 mg CAT/g under DCFA-BG. This relative stability suggests that the quantified flavonoids may have been more resistant to the drying conditions or that degradation losses were partially compensated by increased extractability after dehydration. In berries, drying can disrupt cellular structures and facilitate the release of certain bioactive compounds, although it may also degrade thermolabile molecules such as anthocyanins and other heat-sensitive polyphenols [40,41].

Antioxidant capacity in *V. corymbosum* increased after drying, from 39.18 µmol TE/g in fresh fruit to 66.00 µmol TE/g under DCFA and 63.13 µmol TE/g under DCFA-BG. This increase, despite the partial reduction in total phenolics, may be associated with the concentration of antioxidant compounds due to water loss, improved extractability of intracellular compounds, or the formation of thermal reaction products with reducing capacity. Moreover, antioxidant capacity determined by the DPPH assay does not necessarily correlate directly with total phenolic content, since radical-scavenging activity depends not only on the concentration of phenolic compounds but also on their molecular structure, hydrogen-donating ability, and interactions with other antioxidant constituents present in the extract. Therefore, changes in antioxidant capacity may occur independently of variations in total phenolic content. However, this response should be interpreted with caution, since antioxidant capacity measured by the DPPH assay depends not only on the total amount of phenolics but also on the chemical structure and radical-scavenging reactivity of the compounds present in the extract [19].

In *P. peruviana*, the effect of drying was markedly different. Total phenolic content decreased from 95.73 mg GAE/g in fresh fruit to 43.87 mg GAE/g under DCFA and 45.93 mg GAE/g under DCFA-BG. Similarly, total flavonoid content decreased from 79.44 mg CAT/g to 39.97 and 43.05 mg CAT/g under DCFA and DCFA-BG, respectively. These reductions indicate that the bioactive compounds of goldenberry were more sensitive to dehydration than those of blueberry.

The loss of phenolics and flavonoids in *P. peruviana* may be related to oxidation reactions, thermal degradation, and structural changes occurring during drying. Goldenberry contains antioxidant compounds such as vitamin C, carotenoids, and polyphenols, some of which are sensitive to heat, oxygen, and prolonged processing exposure. Therefore, the reductions observed suggest that drying conditions should be optimized to improve the retention of bioactive quality in this species. Antioxidant capacity in *P. peruviana* showed a substantial decrease after drying, from 94.05 µmol TE/g in fresh fruit to 2.93 µmol TE/g under DCFA and 8.26 µmol TE/g under DCFA-BG. Although both treatments strongly reduced antioxidant activity, DCFA-BG retained higher values than DCFA. This result may be associated with greater thermal stability in the hybrid system or with differences in moisture removal dynamics. However, the magnitude of the reduction indicates that the *P. peruviana* matrix was highly sensitive to the dehydration process.

The contrasting behavior between both species demonstrates that the response of bioactive compounds during drying cannot be generalized across berry-type fruits. In *V. corymbosum*, drying increased antioxidant capacity, possibly due to concentration effects and improved extractability of antioxidant compounds. In contrast, in *P. peruviana*, drying substantially reduced phenolics, flavonoids, and antioxidant capacity, evidencing greater susceptibility of its bioactive matrix to the thermal conditions of the process. When comparing the drying systems, DCFA-BG showed contrasting effects. In *V. corymbosum*, this system resulted in lower total phenolic content than DCFA, although antioxidant capacity remained similar between the two drying treatments. In *P. peruviana*, DCFA-BG preserved antioxidant capacity slightly better than DCFA, but it did not prevent the overall reduction in bioactive compounds compared with fresh fruit. These findings indicate that biogas thermal assistance may improve operational stability during drying, but its effect on bioactive quality depends on the fruit species and should be optimized according to the thermal sensitivity of each product. Therefore, the selection and optimization of solar drying conditions should be species-specific, balancing moisture reduction, product stability, and retention of antioxidant potential.

## 4. Conclusions

Forced-air solar drying and biogas-assisted hybrid solar drying significantly modified the overall quality of *V. corymbosum* and *P. peruviana*. Both systems effectively reduced moisture content and water activity, improving the stability of the dehydrated fruits. The DCFA-BG system showed the best performance in reducing water activity, particularly in blueberry, where it achieved the lowest final moisture content and $a_{w}$ value.

Dehydration increased titratable acidity, total soluble solids, and firmness in both species as a result of solute concentration and tissue shrinkage. However, it also caused visible color changes, especially in *P. peruviana*, which showed greater loss of lightness and reduction in its characteristic yellow hue.

Regarding bioactive properties, *V. corymbosum* showed better functional stability, maintaining flavonoid content and increasing antioxidant capacity after drying. In contrast, *P. peruviana* was more sensitive to dehydration, showing marked reductions in total phenolics, flavonoids, and antioxidant capacity.

Biogas-assisted hybrid solar drying represents a promising technological alternative for improving the stability of dehydrated Andean berries. However, its application should be optimized according to the fruit matrix, balancing moisture reduction, color preservation, bioactive compound retention, and instrumental texture.

The findings of this study provide scientific and technological evidence for optimizing solar drying systems for Andean berries according to the characteristics of each fruit species. Furthermore, the evaluated technologies have potential applications in sustainable postharvest processing, contributing to value addition, improved product quality, and the adoption of renewable energy-based drying systems in small-scale agroindustrial production.

## Figures and Tables

**Table 1:** Experimental design and evaluated treatments.

Factor	Level/Code	Description	Evaluated Variables
Fruit species	*V. corymbosum*	Blueberry	All quality variables
Fruit species	*P. peruviana*	Goldenberry	All quality variables
Treatment condition	FSD	Fresh non-dehydrated fruit; initial control	Physicochemical, bioactive, and textural variables
Treatment condition	DCFA	Forced-air solar drying	Physicochemical, bioactive, textural, and rehydration variables
Treatment condition	DCFA-BG	Biogas-assisted forced-air hybrid solar drying	Physicochemical, bioactive, textural, and rehydration variables

**Table 2:** Technical characteristics of the evaluated solar dehydration systems.

Component	Technical Characteristic
Collector type	Flat-plate solar collector
Absorber plate	Steel, 1050 × 600 × 2 mm, painted black
Thermal insulation	20 mm expanded polystyrene covered with aluminum foil
Glazing	5 mm single glass
Distance between glazing and absorber plate	150 mm
Collector orientation	Static, north-facing, with an approximate azimuth of 20°
Dehydration chamber	1200 × 600 × 900 mm
Chamber cover	2 mm translucent corrugated polycarbonate
Trays	Nylon mesh smaller than 5 mm
Forced-air system	220–240 V axial extractor
Average air mass flow rate	0.038 kg·s^−1^
DCFA system	Forced-air solar drying
DCFA-BG system	Biogas-assisted forced-air hybrid solar drying

**Table 3:** pH, titratable acidity, and total soluble solids of fresh and dehydrated Andean berries.

Species	Treatment	pH	Titratable Acidity (g/100 g)	TSS (°Brix)
*V. corymbosum*	FSD	3.50 ± 0.01 ^ab^	1.00 ± 0.18 ^b^	11.20 ± 1.02 ^c^
	DCFA	3.00 ± 0.06 ^ab^	3.50 ± 0.27 ^ab^	36.70 ± 2.53 ^ab^
	DCFA-BG	3.70 ± 0.10 ^a^	3.60 ± 0.28 ^ab^	33.40 ± 2.93 ^b^
*P. peruviana*	FSD	3.88 ± 0.03 ^b^	1.92 ± 0.10 ^b^	13.90 ± 0.66 ^c^
	DCFA	3.88 ± 0.02 ^b^	4.63 ± 0.28 ^ab^	43.92 ± 1.94 ^a^
	DCFA-BG	4.15 ± 0.18 ^a^	8.50 ± 0.02 ^a^	32.94 ± 1.27 ^b^

Note. Values are expressed as mean ± standard deviation. Different letters within each species and variable indicate significant differences among treatments (*p* < 0.05). FSD: fresh non-dehydrated fruit; DCFA: forced-air solar drying; DCFA-BG: biogas-assisted hybrid solar drying; TSS: total soluble solids.

**Table 4:** Moisture content and water activity of fresh and dehydrated Andean berries.

Species	Treatment	Moisture Content (%)	Water Activity
*V. corymbosum*	FSD	84.95 ± 0.83	0.82 ± 0.01
	DCFA	36.74 ± 0.07	0.73 ± 0.00
	DCFA-BG	22.00 ± 0.19	0.65 ± 0.01
*P. peruviana*	FSD	80.38 ± 0.35	0.79 ± 0.01
	DCFA	24.79 ± 1.11	0.67 ± 0.02
	DCFA-BG	25.16 ± 0.39	0.64 ± 0.01

Note. Values are expressed as mean ± standard deviation. FSD: fresh non-dehydrated fruit; DCFA: forced-air solar drying; DCFA-BG: biogas-assisted hybrid solar drying.

**Table 5:** Instrumental color parameters of fresh and dehydrated Andean berries.

Species	Treatment	(L*)	(a*)	(b*)	(Δ*E*)
*V. corymbosum*	FSD	25.67 ± 0.28 ^a^	0.77 ± 0.08 ^a^	−1.22 ± 0.60 ^c^	—
	DCFA	22.34 ± 0.54 ^ab^	3.72 ± 2.48 ^a^	0.57 ± 0.46 ^b^	4.51 ± 1.69 ^a^
	DCFA-BG	26.45 ± 3.90 ^a^	1.57 ± 0.67 ^a^	2.59 ± 0.61 ^a^	4.90 ± 1.36 ^a^
*P. peruviana*	FSD	63.49 ± 3.60 ^a^	15.72 ± 0.97 ^a^	53.56 ± 5.43 ^a^	—
	DCFA	36.83 ± 1.24 ^b^	20.70 ± 0.76 ^a^	28.54 ± 0.36 ^b^	27.63 ± 2.60 ^a^
	DCFA-BG	34.75 ± 7.00 ^b^	20.96 ± 4.23 ^a^	30.80 ± 7.18 ^b^	37.47 ± 12.43 ^a^

Note. Values are expressed as mean ± standard deviation. Different letters within each species and variable indicate significant differences among treatments (*p* < 0.05). FSD: fresh non-dehydrated fruit; DCFA: forced-air solar drying; DCFA-BG: biogas-assisted hybrid solar drying. (Δ*E*) was calculated using the corresponding fresh fruit as a reference.

**Table 6:** Firmness and rehydration index of fresh and dehydrated Andean berries.

Species	Treatment	Firmness (N)	Rehydration Index
*V. corymbosum*	FSD	2.89 ± 0.10	—
	DCFA	10.14 ± 2.03	2.17 ± 0.39
	DCFA-BG	10.89 ± 2.32	1.94 ± 0.33
*P. peruviana*	FSD	10.89 ± 2.32	1.94 ± 0.33
	DCFA	9.02 ± 0.65	1.91 ± 0.58
	DCFA-BG	6.89 ± 1.19	2.13 ± 0.41

Note. Values are expressed as mean ± standard deviation. FSD: fresh non-dehydrated fruit; DCFA: forced-air solar drying; DCFA-BG: biogas-assisted hybrid solar drying. The rehydration index was determined only in dehydrated samples.

**Table 7:** Bioactive compounds and antioxidant capacity of fresh and dehydrated Andean berries.

Species	Treatment	TPC (mg GAE/g)	TFC (mg CAT/g)	Antioxidant Capacity (µmol TE/g)
*V. corymbosum*	FSD	152.00 ± 0.00 ^a^	107.64 ± 3.20 ^a^	39.18 ± 1.68 ^b^
	DCFA	142.00 ± 7.63 ^ab^	114.85 ± 9.45 ^a^	66.00 ± 0.60 ^a^
	DCFA-BG	128.20 ± 7.41 ^b^	105.87 ± 4.70 ^a^	63.13 ± 1.36 ^a^
*P. peruviana*	FSD	95.73 ± 1.51 ^a^	79.44 ± 3.20 ^a^	94.05 ± 3.19 ^a^
	DCFA	43.87 ± 3.91 ^b^	39.97 ± 1.18 ^b^	2.93 ± 0.65 ^c^
	DCFA-BG	45.93 ± 2.37 ^b^	43.05 ± 3.47 ^b^	8.26 ± 0.50 ^b^

Note. Values are expressed as mean ± standard deviation. Different letters within each species and variable indicate significant differences among treatments (*p* < 0.05). FSD: fresh non-dehydrated fruit; DCFA: forced-air solar drying; DCFA-BG: biogas-assisted hybrid solar drying; TPC: total phenolic content; TFC: total flavonoid content; GAE: gallic acid equivalents; CAT: catechin equivalents; TE: Trolox equivalents.

## Data Availability

The study’s original contributions are included in the article. Further inquiries can be directed to the corresponding authors.
